# Can a Fully Electronic Patient Record-Based Virtual Fracture Clinic Increase Compliance With British Orthopaedic Association Guidelines for the Assessment of Acute Orthopaedic Trauma Patients?

**DOI:** 10.7759/cureus.46942

**Published:** 2023-10-13

**Authors:** Abdelwakeel Bakhiet, William Passmore, Upamanyu Nath, Abhirun Das, Anand Pillai

**Affiliations:** 1 Trauma and Orthopaedics, Wythenshawe Hospital, Manchester University National Health Service (NHS) Foundation Trust, Manchester, GBR; 2 Trauma and Orthopaedics, University of Manchester, Manchester, GBR

**Keywords:** virtual fracture clinic, electronic patient record, epr, hive, vfc

## Abstract

Introduction

There is clear guidance for the follow-up of acute orthopaedic trauma patients within 72 hours of presentation as per British Orthopaedic Association (BOA) guidelines. The virtual fracture clinic (VFC) model has been adopted nationwide to increase compliance with these guidelines. Traditionally our VFC was paper-based, but recently it has been made completely electronic with the introduction of Hive which is a comprehensive electronic patient record (EPR). The aim of this audit was to assess the effectiveness of the Hive VFC and to see the impact it has on improving the patient experience.

Methods

Data was collected retrospectively by looking at referrals to the Hive VFC across a seven-month period from its date of introduction. No patients were excluded and data was collected for 2,158 patients. Patient demographics, referral details, and outcomes were recorded on a Microsoft Excel version 16.0 for Windows spreadsheet (Microsoft Corporation, Washington, United States). Data was analysed using Microsoft Excel.

Results

Data was collected for 2,158 patients between September 9, 2022, and March 31, 2023, in the Hive VFC. The largest proportion of injuries was found in the foot and ankle region with 32% of referrals (n=688), followed by hand and wrist with 30% (n=651), upper limb with 28% (n=595), and lower limb with 7% (n=142). There was a 50.4% (n=125) increase in the number of patients seen in the VFC across the study period with 248 patients being seen in September 2022, increasing to 373 in March 2023. The number of patients with acute traumatic orthopaedic injuries that were assessed within 72 hours on average was 94.5% per month. There was an increase of 7.3% during the study period from September 2022 to March 2023, 89.9% to 96.5%, respectively.

Conclusion

We believe that Hive VFC is a clinically sound and effective way of assessing acute traumatic orthopaedic patients and increasing compliance with BOA and NICE guidelines. The number of patients needing acute orthopaedic assessment is rising steadily, and this should alert us to find more convenient, time and cost-saving methods of delivering safe and effective patient care.

## Introduction

There is clear guidance for the follow-up of acute traumatic orthopaedic injury patients published by the British Orthopaedic Association (BOA) stating that ‘patients should be seen in a new fracture clinic within 72 hours of presentation with the injury’ [1]. However, due to the ever-increasing demand for trauma services and increasing numbers of accident and emergency attendances, clinics have become overbooked, often with patients that do not require any orthopaedic intervention [2]. This has led to longer waiting times for patients, overstretched healthcare professionals, and delays in secondary care for those who require it [3].

The virtual fracture clinic (VFC) was first introduced as a concept by the Glasgow Royal Infirmary in 2011 [4]. The VFC allows initial management, imaging, and triage to take place in the emergency department, which is then supplemented by a further remote assessment of suitable injuries and fractures online [5]. The VFC is made up of a team led by a consultant orthopaedic surgeon, a nurse, and administrative staff. The referrals are all added to a clinic list, and the images and clinical notes should be readily available. The cases are reviewed individually and assigned to one of three outcomes: review in the general clinic, review in the specialty clinic, or discharge with advice. The outcome should then be conveyed to the patient and general practitioner (GP) via letter, and the patient may be directed to useful resources such as leaflets or rehabilitation services [4].

Since its coining, the VFC has been adopted nationwide with over 40 units having implemented its principles [5]. There have been numerous studies that have established the VFC as a cost-effective and efficient way of increasing adherence to the BOA and National Institute for Health and Care Excellence (NICE) standards [6]. It has also been shown to improve patient satisfaction, through reduced unnecessary face-to-face appointments, and improve patient safety with fewer cases of patients not attending their follow-up clinics [7].

Traditionally in our trust, the VFC was a paper-based system, but recently, it has been moved to a completely electronic system. Hive is a digitally advanced electronic patient record (EPR) that was introduced at Manchester University Foundation Trust (MFT) in September 2022 and aims to synchronise all of MFT’s sites allowing access to one shared patient record [8]. Hive is a variant of the EPIC system which is the largest EPR in the world and allows clinicians and patients access to a single comprehensive medical record. This reduces the need to repeat information across different systems and reduces the amount of time spent by staff gathering information [9]. The aim of this audit was to assess the effectiveness of the Hive VFC and to see the impact it has on improving the patient experience.

## Materials and methods

We conducted a cross-sectional study looking at referrals to the VFC following the implementation of Hive at Wythenshawe Hospital, Manchester, United Kingdom. We retrospectively collected data from prospectively recorded data on the Hive EPR between September 9, 2022, and March 31, 2023. Using Hive, we analysed data for 2,158 patients. Hive is a comprehensive EPR that allows comprehensive access to all patient history, notes, images, investigations, previous letters, and final VFC outcomes. We collected data on the patient's age, gender, anatomical location of injuries, time of admission, waiting time for VFC, and time of discharge.

This study includes all of the patients referred to the VFC in our hospital between September 9, 2022, and March 31, 2023. This includes all referrals from the emergency department, local general practices, and minor injuries units. There were no exclusions.

Data was collated within Microsoft Excel version 16.0 for Windows (Microsoft Corporation, Washington, United States). We generated master tables including the data points of interest, date of injury, number of days from referral to review, review outcome, and clinician responsible for the patient care. The data was then analysed within Microsoft Excel.

We used NICE, BOA standards for trauma (BOAST 7) fracture management guidelines and locally agreed trust protocols as a standard of reference for this study [1,10].

## Results

In total, 2,158 patients were seen between September 9, 2022, and March 31, 2023, in the Hive VFC. The gender distribution of the patients who were assessed was 47% male and 53% female (1,015 and 1,143, respectively, n=2158).

The ages of the patients assessed in the VFC were also recorded. The majority of patients were ages 18-65 with 52% of patients being in this category (n=1,123). This was followed by ages 0-18 with 30.9% and ages above 65 with 17.1% of injuries (n=666 and 369, respectively).

**Figure 1:**
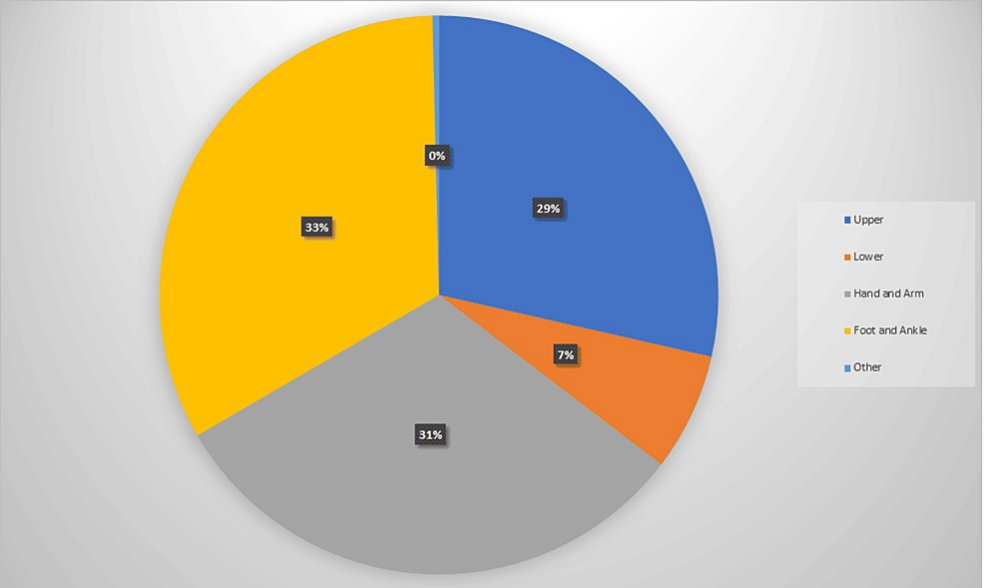
The distribution of anatomical locations in the Hive VFC

To further understand the distribution of injuries as per anatomical location, cases referred to the VFC were stratified in groups according to subspecialty. The largest proportion of injuries was found in the foot and ankle region with 32% of referrals, 30% were hand and wrist, 28% were upper limb, and 7% were lower limb (N=688, 651, 595, and 142, respectively). Unfortunately, documentation was unclear in 3% of cases (n=82) (Figure 1).

**Figure 2:**
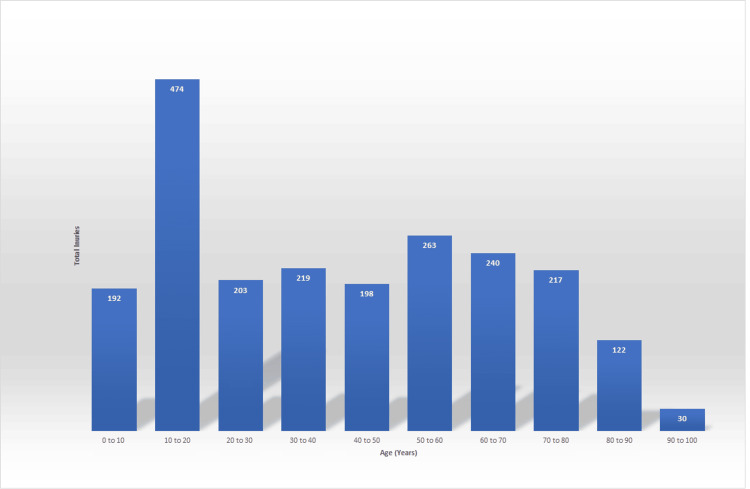
The number of patients assessed per month during the study period

Across the study period, a mean number of patients were seen daily in the VFC of 10.6. The number of patients assessed in the VFC per month was 248 in September 2022, 285 in October, 269 in November, 309 in December, 321 in January, 353 in February, and 373 in March 2023. This illustrates a 50.4% increase in the volume of patients assessed in the VFC across the study period (n=125) (Figure 2).

The number of patients with acute traumatic orthopaedic injuries that were assessed within 72 hours on average was 94.5% per month. There was an increase of 7.3% during the study period from September 2022 to March 2023, 89.9% to 96.5%, respectively. This demonstrates improved compliance with the BOAST 7 guidelines.

All patients were assigned a named consultant and were seen by senior orthopaedic staff or GPs who were directly supervised by an orthopaedic consultant. All consultations were completed with a clear management plan that was communicated to both the patient and the GP via letter.

## Discussion

We are now seeing more patients in VFC than ever before, with an obvious trend of more units adopting this virtual model for assessing acute traumatic orthopaedic injuries [11]. Although initially after the VFC model was first introduced, many were reluctant to adopt an online virtual system for the NHS. This was due to worries about duplicated work and fears of the risk of a data breach and its potential consequences. However, with the availability of newer technologies such as Hive, these systems allow a safer more stringent way of implementing this model and improving patient care [12].

Through looking at our conclusive data series of 2,158 patients referred across the six-month period to Wythenshawe Hospital’s Hive VFC, we were able to analyse different parameters and confirm that the Hive VFC is an effective and clinically safe manner of assessing acute traumatic orthopaedic presentations. Through stratifying the data based on the anatomical location of the injury, we have shown that the highest number of patients were amongst foot and ankle and hand and wrist patients. These injuries are well known to be time-sensitive as poor or delayed treatment can lead to reduced function and joint stiffness [3]. However, the Hive VFC allows patients to be treated in a timely manner and appropriately directed to the appropriate specialty, thus reducing unnecessary delays [12].

The number of patients seen in the Hive VFC per month saw a 50.4% increase from the beginning of the study period to the end. This could be due to improved awareness amongst referring clinicians utilising the HIVE VFC as well as increased confidence from those running the clinic as they become more familiar with the system. This is a similar finding to studies that investigated the effects of a newly introduced VFC and found attendance rates to improve by 56.4% during their first six months [13].

When reviewing the literature, an important effect of introducing the VFC system in the United Kingdom seems to be improving compliance with BOA and NICE guidelines for the assessment of acute traumatic orthopaedic patients within 72 hours [14]. This study illustrates that the Hive VFC is an effective way of further increasing the efficiency of patients being assessed through a 7.33% rise in patients seen within 72 hours of presentation. This leads to an improved patient experience, improved discharge rates, and reduced wasted face-to-face appointments. The time saved from this could be applied to improve standards of patient care in other domains through teaching and training. There were no noted instances of patients missing a necessary face-to-face consultation due to their VFC outcome.

The data collected in this study was conclusive over a seven-month period, including 2,158 patients with no patients being excluded. This is a strength in favor of the findings and suggests that these are representative. The completely digital system undoubtedly made it easier to access patient data without restrictions and reduced inconsistencies associated with paper documentation.

However, due to the study being retrospective, data collection was dependent on the accuracy of previous documentation and interaction. This highlights the possibility of inaccurate or inconsistent information or misinterpretation of clinical findings by emergency department staff. Considering that those collecting the data had no control over the process of documentation, some limitations may lie therein.

We believe that further work to investigate the impact of a digitalised VFC would be useful in investigating the effects of VFC on patient outcomes. This could take reattendances, representation to emergency services, and patient satisfaction into account. A further study looking into the accuracy of the VFC would be useful by following up with patients and re-examining them to confirm the previous findings and also detect possible complications.

## Conclusions

We believe that Hive VFC is a clinically sound and effective way of assessing acute traumatic orthopaedic patients and increasing compliance with BOA and NICE guidelines. The use of a fully EPR-based VFC aids in assessing patients comprehensively and efficiently. The use of this robust technology provides better patient service and should be embraced proactively. The number of patients needing acute orthopaedic assessment is rising steadily, and this should alert us to find more convenient, time and cost-saving methods of delivering safe and effective patient care.
